# Nicotine Therapy for Parkinson’s Disease: A Meta-Analysis of Randomized Controlled Trials

**DOI:** 10.3390/biomedicines13081814

**Published:** 2025-07-24

**Authors:** Chih-Hung Liang, Tsai-Wei Huang, Wei-Ting Chiu, Chen-Chih Chung, Chien-Tai Hong

**Affiliations:** 1 Department of Neurology, Shuang-Ho Hospital, Taipei Medical University, New Taipei City 23561, Taiwan; 23347@s.tmu.edu.tw (C.-H.L.); 11440@s.tmu.edu.tw (W.-T.C.); 2 Cochrane Taiwan, Taipei Medical University, Taipei 11031, Taiwan; tsaiwei@tmu.edu.tw; 3 School of Nursing, College of Nursing, Taipei Medical University, Taipei 11031, Taiwan; 4 Department of Neurology, School of Medicine, College of Medicine, Taipei Medical University, Taipei 11031, Taiwan

**Keywords:** Parkinson’s disease, nicotine, randomized controlled trial, motor symptoms, meta-analysis

## Abstract

**Background:** Epidemiological studies have reported an inverse association between smoking and Parkinson’s disease (PD) risk, prompting interest in nicotine as a potential therapeutic agent. The present meta-analysis evaluated the efficacy of nicotine therapy in improving motor symptoms and activities of daily living in patients with PD. **Methods:** PubMed, Embase, and Cochrane Library were systematically searched to identify randomized controlled trials (RCTs) assessing nicotine therapy in PD. Clinical RCTs administering interventions extending beyond 1 week and reporting motor or nonmotor outcomes were included. Random-effects models were used to analyze short-term (<6 months) and long-term (≥6 months) outcomes by using standardized mean differences (SMDs). **Results:** This meta-analysis included five RCTs (346 participants). Nicotine therapy led to no significant improvement in motor outcomes in the short term (pooled SMD: −0.452, 95% confidence interval: −1.612 to 0.708) or long term (pooled SMD: 0.174, 95% confidence interval: −0.438 to 0.787). Considerable interstudy heterogeneity was noted. Furthermore, short-term nicotine therapy resulted in no significant improvement in daily functioning, cognition, or quality of life. **Conclusions:** This meta-analysis revealed a lack of compelling evidence suggesting that nicotine-based therapies improve motor or nonmotor outcomes in PD. The findings highlight a disconnect between epidemiological associations and clinical efficacy. Given the prodromal nature of PD pathology and the challenges of early diagnosis, future preventive strategies should be implemented before symptom onset in high-risk individuals identified using advanced biomarker panels.

## 1. Introduction

Parkinson’s disease (PD) is a progressive neurodegenerative disorder characterized by motor symptoms, such as tremor, bradykinesia, rigidity, and postural instability, and nonmotor symptoms that impair daily functioning [1]. Mainstream therapies for PD, such as the use of levodopa and dopaminergic agonists, can effectively relieve symptoms. However, to the best of our knowledge, none of the available PD therapies can modify the disease course [2]. To address this gap, numerous large-scale randomized controlled trials (RCTs) have assessed the disease-modifying potential of candidate treatments that yielded promising results in in vitro, in vivo, and early-stage clinical trials [3]. However, nearly all of these candidate treatments, including those involving coenzyme Q10 [4], pioglitazone [5], and exenatide [6], have failed to exert substantial disease-modifying effects. Consequently, the search for a viable disease-modifying therapy for PD continues.

Epidemiological evidence suggests that cigarette smoking is associated with a relatively low risk of PD [7]. A meta-analysis [8] and a large-scale cohort study [9] have demonstrated an inverse association between cigarette smoking and PD risk. Nicotine, a cholinergic agonist, has been proposed to be the component responsible for the neuroprotective and dopaminergic-modulating benefits of cigarette smoking. In the context of PD, nicotine exerts its neuroprotective effects primarily by activating nicotinic acetylcholine receptors, which enhance dopamine release, reduce oxidative stress, and inhibit neuroinflammation. Moreover, nicotine stimulates intracellular signaling cascades such as the phosphoinositide 3-kinase/protein kinase B and extracellular signal-regulated kinase pathways, promoting neuronal survival, supporting mitochondrial function, and suppressing α-synuclein aggregation [10].

Research indicates that nicotine replacement therapy (NRT) not only confers neuroprotection but also alleviates the motor symptoms of PD. Kelton et al. reported that acute nicotine infusion and 2-week transdermal nicotine patch application improved motor performance in patients with PD [11]. However, an open-label trial of nicotine patch therapy revealed poor tolerability and no motor symptom relief [12].

Overall, the effects of nicotine against PD remain unclear. Nicotine can be delivered through various methods, such as intragum administration, patch application, and dietary supplements. Although several RCTs have evaluated the efficacy of nicotine in managing PD, their findings remain to be systematically reviewed and synthesized in a meta-analysis. Thus, the present meta-analysis was conducted to comprehensively investigate the effects of nicotine on PD motor symptoms, aiming to determine its therapeutic potential and inform future clinical practice.

## 2. Materials and Methods

### 2.1. Search Strategy and Study Selection

The study protocol adhered to the Preferred Reporting Items for Systematic Reviews and Meta-Analyses guidelines [13]. PubMed, Embase, and Cochrane Library were systematically searched for relevant articles published till October 2024. The search strategy was as follows: “NRT OR nicotine” AND “Parkinson” AND “Randomized controlled trial.” No language restrictions were applied. After the removal of duplicates, titles and abstracts were screened for relevance. Potentially eligible articles were subjected to full-text review on the basis of predefined selection criteria.

We included RCTs (including those with a double-blind and open-label randomized design) that investigated the therapeutic effects of ≥1-week-long nicotine use or nicotine-based interventions (e.g., transdermal nicotine patches, oral nicotine formulations, and nicotine-rich diets) versus placebo or control treatment on motor symptoms or nonmotor aspects (e.g., activities of daily living [ADLs], cognition, or quality of life [QoL]) in patients with PD. We excluded studies that were not RCTs, did not include a PD control group, or lacked relevant outcome data.

### 2.2. Data Extraction and Quality Assessment

Two reviewers (CH Liang and CT Hong) independently extracted data from each included study by using a standardized form. The following data were extracted: study characteristics (authors, publication year, country, and design), cohort details (sample size, disease duration, PD severity, and concurrent medications), intervention details (nicotine form, nicotine dose, and treatment duration), control conditions, and outcome measures (motor function, ADL performance, cognition, and QoL). The reviewers independently performed screening and data extraction. Between-reviewer discrepancies were resolved through discussion and, if required, consultation with a third reviewer until a consensus was reached. Risk of bias was evaluated using the Cochrane Risk-of-Bias 2 tool [14]. Publication bias could not be assessed because of the small sample size.

### 2.3. Outcomes and Statistical Analysis

The following outcomes were analyzed: motor symptom severity, ADL performance, QoL, and cognition. Both short-term (treatment duration: <6 months) and long-term (treatment duration: 6–12 months) outcomes were investigated. Motor symptoms were assessed using the Unified Parkinson’s Disease Rating Scale (UPDRS) Part III or the total UPDRS (Parts I–III) at baseline and immediately after intervention. ADL performance was evaluated using UPDRS Part II or other validated ADL scales, such as the Schwab and England scale. QoL was assessed using two versions of the Parkinson’s Disease Questionnaire. Finally, cognition was measured using the Mattis Dementia Rating Scale and the Scales for Outcomes in Parkinson’s Disease–Cognition.

Considering the high likelihood of heterogeneity in study cohort and intervention, we conducted the meta-analysis by using random-effects models based on the DerSimonian–Laird method. When different scales or metrics were used for a given outcome across studies, we calculated standardized mean differences (SMDs) with 95% confidence intervals (CIs) for data pooling. Heterogeneity was assessed using the *I*^2^ statistic and chi-square test. *I*^2^ values of >50% indicated substantial heterogeneity. All analyses were performed using OpenMeta[Analyst] (v5.26.14, Brown University, Providence, RI, USA). All tests were two-tailed, and a *p*-value of <0.05 indicated statistical significance. The meta-analysis results for primary outcomes are depicted in forest plots. Pooled effect sizes with corresponding CIs and *p*-values are reported. The results of individual studies are presented to facilitate the interpretation and contextualization of the pooled findings.

## 3. Results

### 3.1. Summary of Included Studies

**Figure 1:**
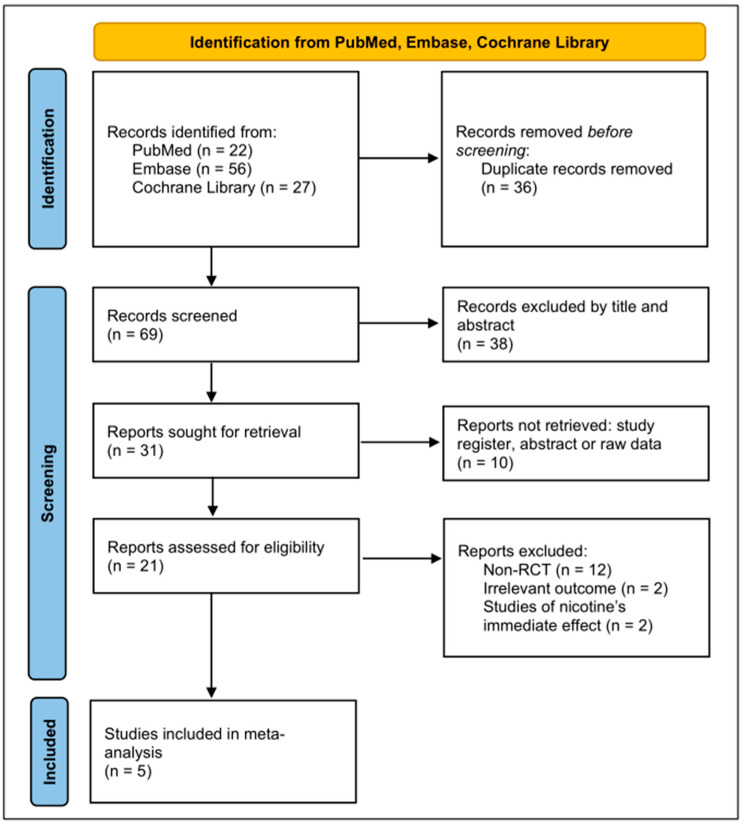
Preferred Reporting Items for Systematic Reviews and Meta-Analyses flowchart depicting the study selection process.

**Figure 2:**
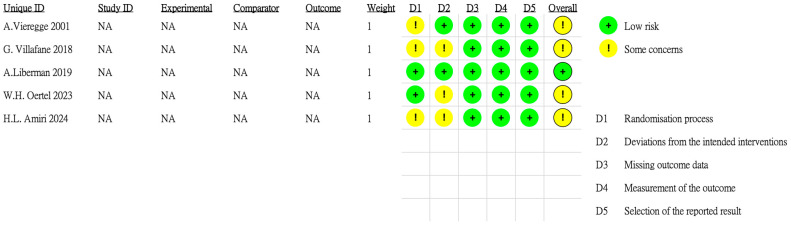
Results of study quality assessment [15,16,17,18,19].

Figure 1 depicts the study selection process. The literature search yielded 105 articles. After the removal of 36 duplicates, 69 unique articles were screened. Of these articles, 38 were excluded after title or abstract screening. The remaining 31 full-text articles were assessed for eligibility, and 26 were excluded for reasons such as not being an RCT, having a nonrandomized design, and lacking relevant outcome data. Finally, five RCTs were included in both qualitative and quantitative analyses. Quality assessment revealed that one RCT had a low risk of bias because of its placebo-controlled, double-blind design, whereas four RCTs had some concerns regarding bias (Figure 2).

The included RCTs varied in terms of design, cohort, nicotine intervention, and outcome measures (Table 1) [15,16,17,18,19]. Regarding study design, three RCTs were double-blind, placebo-controlled trials; one was an open-label trial with blinded outcome evaluation; and one was a nonblinded trial of dietary intervention. Regarding the study cohort, the included RCTs ranged from small single-center trials (32–45 patients) to a large multicenter trial focusing on early-stage PD (163 patients across 24 sites). Notably, disease stage varied across the cohorts; some RCTs enrolled patients with early-stage, de novo PD (Hoehn and Yahr stage I or II), whereas others included patients with relatively advanced disease (Hoehn and Yahr stage IV). Regarding nicotine intervention, multiple RCTs tested high-dose transdermal nicotine patches, one RCT tested oral nicotine capsules, and one RCT tested a nicotine-enriched diet. Treatment duration varied across the RCTs, ranging from approximately 10–12 weeks in short-term RCTs to >6–12 months in long-term RCTs. All RCTs primarily evaluated motor symptoms. Some RCTs additionally evaluated ADL performance, along with other clinical parameters. For example, one RCT focused on levodopa-induced dyskinesia, whereas other RCTs assessed gait-related outcomes, such as falls, freezing of gait, or biomarker (e.g., α-synuclein) levels, along with standard motor measures. Although study design and specific objectives varied across the RCTs, all five trials investigated whether nicotine-based interventions ameliorate motor symptoms or decelerate PD progression.

### 3.2. Changes in Motor Symptoms

**Figure 3:**
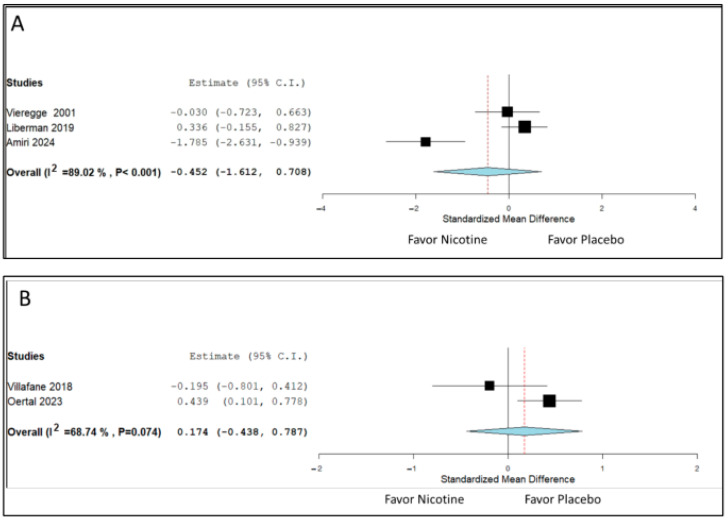
Effects of nicotine therapy on the motor symptoms of patients with Parkinson’s disease. (**A**) Results from studies with treatment durations of less than 6 months [15,16,19]. (**B**) Results from studies with treatment durations of 6–12 months [17,18]. Horizontal lines represent 95% confidence intervals (CIs) for the standardized mean difference. Negative values indicate postintervention amelioration of motor symptoms. Diamonds represent pooled effect estimates. High heterogeneity was observed among the studies (*I*^2^ = 89% for short-term outcomes and 68% for long-term outcomes), indicating substantial variability, likely attributable to interstudy differences in nicotine dose, disease stage, and delivery method.

Three RCTs provided data on the motor outcomes of short-term nicotine therapy. The pooled SMD value was −0.452 (95% CI: −1.612 to 0.708), indicating that nicotine therapy led to no significant improvements in motor outcomes compared with the effects of placebo (Figure 3A). Substantial interstudy heterogeneity was observed (*I*^2^ = 89%). Two RCTs provided data on the motor outcomes of long-term nicotine therapy. The pooled SMD value was 0.174 (95% CI: −0.438 to 0.787), indicating no significant benefits from nicotine therapy. Substantial heterogeneity was noted (*I*^2^ = 68%; Figure 3B).

### 3.3. Changes in ADLs, QoL, and Cognition

**Figure 4:**
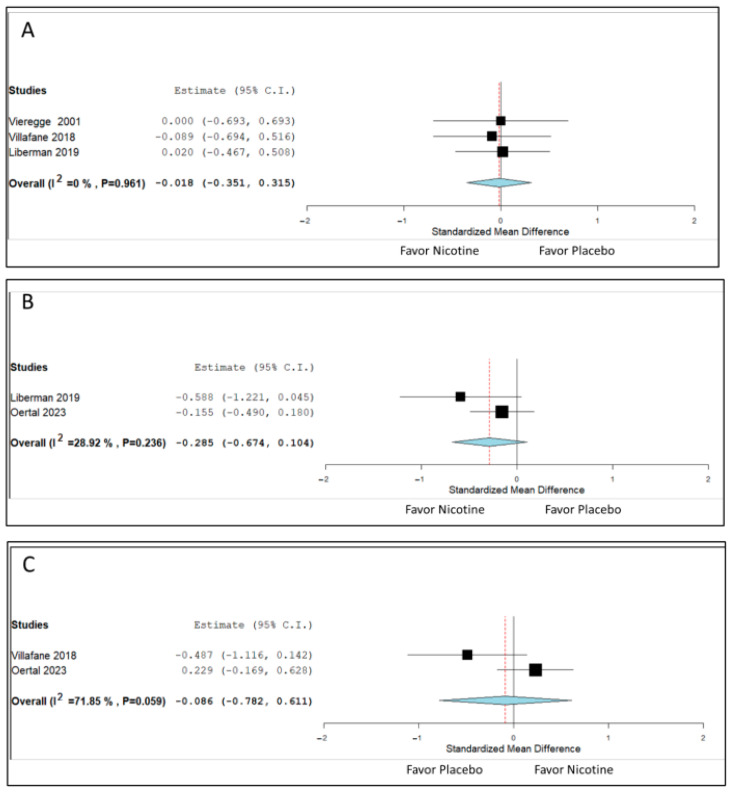
Effects of nicotine therapy on nonmotor aspects in patients with Parkinson’s disease. (**A**) Activities of daily living, measured primarily using the Unified Parkinson’s Disease Rating Scale Part II [15,18,19]; (**B**) quality of life, assessed using the 39-item Parkinson’s Disease Questionnaire [15,17]; and (**C**) cognition, measured using validated scales such as the Mattis Dementia Rating Scale [17,18]. Standardized mean differences and 95% confidence intervals (CIs) are presented for each study and pooled estimate. Symbols represent individual study outcomes, and diamonds represent pooled effects. Minimal heterogeneity was observed for activities of daily living outcomes (*I*^2^ = 0%), whereas moderate to high heterogeneity was observed for quality of life and cognition, likely attributable to interstudy differences in study design and instrument.

Three RCTs reported ADL outcomes. The pooled SMD value was −0.018 (95% CI: −0.351 to 0.315), indicating no significant difference between the nicotine and placebo groups. Heterogeneity was minimal (*I*^2^ = 0%; Figure 4A). In long-term studies, nicotine therapy led to no significant improvement in QoL (primarily assessed using the 39-item Parkinson’s Disease Questionnaire; Figure 4B) or cognition (Figure 4C).

## 4. Discussion

Epidemiological studies have consistently indicated that smoking reduces the risk of PD. Thus, researchers have explored the potential use of nicotine for neuroprotection and disease modification. However, relevant clinical evidence is inconsistent. To the best of cour knowledge, this meta-analysis is the first to analyze the effects of various nicotineccbased interventions on motor function and ADLs in patients with PD. We reviewed five RCTs published between 2001 and 2024 to determine the therapeutic potential of nicotine-based interventions. The pooled results indicate that short-term administration of nicotine through patches, oral formulations, or dietary sources did not significantly reduce motor severity or improve ADL performance. Even long-term administration (>1 year) did not decelerate motor progression. These findings are consistent with those of the largest clinical trial on this subject to date, the NIC-PD study [17], which reported no benefits from transdermal nicotine patch application and slightly poorer outcomes in the intervention group than in the placebo group. Our findings suggest that although epidemiological and preclinical data have been encouraging, nicotine’s therapeutic potential against PD has not yet been supported by clinical trials.

Among the included RCTs, two indicated that conventional outcome measures such as UPDRS Part III scores do not capture all potential benefits of nicotinic agents. Villafane et al. [18] and Liberman et al. [15] reported potential secondary benefits of nicotine use. Although Villafane et al. [18] observed that UPDRS-III scores did not differ significantly between the intervention and control groups, they noted potential improvements in daily functioning during the OFF stage (UPDRS Part II), motor fluctuations (UPDRS Part IV), and reductions in the required dosage of dopaminergic medication. Considering their study’s open-label design and potential placebo effects, they interpreted the aforementioned findings with caution. Liberman et al. [15] revealed no substantial improvement in motor function with nicotine use. Notably, the placebo group exhibited a more favorable trend than did the intervention group. However, a reanalysis of secondary outcomes indicated considerably lower incidence of falls and freezing of gait in patients who received oral nicotine for 10 weeks than in those who received placebo. These findings were attributed to nicotine’s direct action on nicotinic acetylcholine receptors located in the pedunculopontine nuclei. However, the aforementioned outcomes were reported in single studies and have not been replicated in other RCTs. Therefore, although intriguing, the outcomes were isolated findings. Additional trials are required to determine whether nicotine can consistently mitigate the motor and nonmotor symptoms of PD.

Villafane et al. [18] reported postintervention improvements in secondary outcomes, which were not noted in other RCTs. The intertrial differences may be explained by variability in disease stage and nicotine dose. Villafane et al. enrolled patients with relatively advanced PD (with Hoehn and Yahr stages up to IV) and administered (through transdermal patches) nicotine at a high dose (up to 90 mg/day). By contrast, Oertel et al. [17] and Vieregge et al. [19] included patients with early-stage PD (Hoehn and Yahr stages of ≤2 and ≤3, respectively) and administered (through transdermal patches) nicotine at a low dose (up to 35 mg/day). The intertrial differences in outcomes may be attributable to interstudy variability in disease stage, nicotine dose, and delivery method. Therefore, clinicians should consider patient characteristics and dosing strategies when interpreting efficacy signals. Patients with advanced PD often present with extensive cholinergic deficits, which may partly explain the observed intertrial differences in the effects of nicotinic receptor stimulation, particularly those against nondopaminergic symptoms and motor complications. Higher doses administered through nicotine patches resulted in greater absorption and higher bloodstream concentrations [20]. The bioavailability of oral nicotine (NC001), used in the study of Liberman et al. [15], and dietary nicotine, used in the study of Amiri et al. [16], remains unclear. Evidence suggests that oral NRT is less potent than transdermal NRT [21,22]. Overall, a high-dose regimen can boost the pharmacologic effects of nicotine, surpassing the threshold required for achieving functional improvements beyond symptomatic relief. The aforementioned findings indicate that both disease severity and dosage intensity should be considered when determining the efficacy of nicotine in PD management.

Research has highlighted the protective effect of cigarette smoking against PD, but this effect may be attributable to the characteristics of smokers. A preliminary study indicated that smoking itself does not confer neuroprotection; rather, individuals with higher intrinsic dopamine levels may be more prone to addiction and less susceptible to PD [23]. Certain personality traits, such as neuroticism and introversion, have been associated with an increased risk of PD. Evidence suggests that smoking is associated with personality traits such as low neuroticism, which is associated with a reduced risk of PD [24]. However, the direction and nature of such an association remain unclear. Most of the proposed mechanisms underlying the protective effects of cigarette smoking are indirect and speculative, which may explain why these effects did not translate into therapeutic benefits. Our meta-analysis revealed a discrepancy between epidemiological evidence on the protective effects of cigarette smoking and the limited efficacy of nicotine in RCTs. The temporal nature of PD pathogenesis should also be considered. A growing body of evidence indicates that the neurodegenerative process begins decades before the onset of clinical motor symptoms, with substantial neuronal loss already present at the time of diagnosis [25]. Given the extended prodromal phase of PD, initiating disease-modifying therapy only after symptom onset may limit its potential to decelerate disease progression. Notably, by the time PD is clinically diagnosed, a substantial proportion (>50%) of the dopaminergic neurons in the substantia nigra have already been lost, limiting the efficacy of neuroprotective interventions initiated at this stage. Nicotine’s failure to decelerate PD progression, as reported in the NIC-PD trial [17], may reflect the aforementioned problem, highlighting the need for exploring interventions aimed to be implemented during the prodromal or at-risk stages of the disease.

The primary strengths of this meta-analysis are its novelty and the populations studied. We synthesized evidence on the efficacy of different NRT approaches in managing PD. Unlike other studies, which focused primarily on PD risk in the general population, we focused on the therapeutic potential of nicotine in patients with a confirmed diagnosis of PD. Currently, PD is typically diagnosed only after the onset of motor symptoms, given the difficulties in detecting it during the prodromal phase. Thus, all PD-related RCTs, including those investigating NRT, have focused on patients already in the symptomatic stage of the disease. Nonetheless, continual advances in risk prediction algorithms and biomarker panels capable of identifying individuals in the prodromal phase of PD [26,27] may increase the feasibility of administering NRT or other preventive interventions to at-risk populations before substantial neurodegeneration, thereby improving the disease trajectory.

This study has several limitations. The small sample size and the interstudy heterogeneity in design limited the statistical power and generalizability of our findings. Interstudy differences, such as in intervention type (transdermal patches, oral formulations, and dietary intake), cohort (patients with early-stage PD vs. those with advanced PD), and outcome measures, contributed to substantial statistical heterogeneity. Although we used random-effects models to account for the observed variability, the pooled estimates should be interpreted with caution. Another limitation of our study is that the meta-analysis focused primarily on motor symptoms and ADLs, without exploring other nonmotor symptoms such as mood disturbances or sleep disorders. Nicotine may offer some benefits in these aspects, which would, in turn, improve overall QoL in patients with PD.

## 5. Conclusions

This meta-analysis revealed a lack of convincing evidence suggesting that nicotine therapy improves motor function, ADL performance, cognition, and QoL or decelerates disease progression in symptomatic patients with PD. Although preclinical and epidemiological data indicate the therapeutic potential of nicotine, rigorous RCTs do not support its benefits. Future studies should focus on developing selective agents that bind to nicotinic acetylcholine receptors or exploring their benefits when administered during the prodromal phase. In the meantime, research efforts should be directed toward managing known modifiable risk factors for PD by using evidence-based alternatives. Furthermore, clinicians should advise patients that nicotine use, including cigarette smoking, is not a validated or effective treatment option for PD.

## Figures and Tables

**Table 1:** Characteristics of included studies.

Study (First Author, Publication Year)	Country/ Setting	Sample Size	Age (Years), Mean ± Standard Deviation	H and Y Stage	Intervention	Treatment Duration (Weeks)	Outcomes
Vieregge, 2001 [19]	Germany	32	I: 67 ± 6.8 C: 66 ± 7.5	II or III	Transdermal patch, 35 mg/day	3	Motor function and ADL performance
Villafane, 2018 [18]	France	42	I: 58.0 ± 8.4 C: 56.9 ± 6.3	II–IV	Transdermal patch, 90 mg/day	39	Motor function, ADL performance, and cognition
Lieberman, 2019 [15]	United States	65	I: 68.1 ± 8.3 C: 65.5 ± 7.2	II–IV	Oral nicotine bitartrate, 24 mg/day	10	L-Dopa-induced dyskinesia, motor function, and ADL performance
Oertel, 2023 [17]	United States and Germany	162	I: 61.0 ± 9.5 C: 61.0 ± 10.3	I or II	Transdermal patch, 28 mg/day	52	Motor function, cognition, depression, and quality of life
Amiri, 2024 [16]	Iran	45 *	I: 61.53 ± 8.33 C: 60 ± 7.21	Not reported	Nicotine-rich diet, 20 µg/day	12	Motor function and ADL performance

Abbreviations: ADL, activities of daily living; H and Y, Hoehn and Yahr; I, intervention; C, control/placebo. * The original cohort was split into three groups, but we considered only the nicotine group (n = 15) and control group (n = 15).

## Data Availability

The data presented in this study are contained within the manuscript.
